# Spatial prediction of malaria prevalence in an endemic area of Bangladesh

**DOI:** 10.1186/1475-2875-9-120

**Published:** 2010-05-09

**Authors:** Ubydul Haque, Ricardo J Soares Magalhães, Heidi L Reid, Archie CA Clements, Syed Masud Ahmed, Akramul Islam, Taro Yamamoto, Rashidul Haque, Gregory E Glass

**Affiliations:** 1 International Center for Diarrhoeal Disease Research Bangladesh, 68 Shaheed Tajuddin Ahmed Sharani, Mohakhali, Dhaka 1212, Bangladesh; 2 University of Queensland, School of Population Health, Herston, Queensland, Australia; 3 BRAC, BRAC Centre, 75 Mohakhali, Dhaka 1212, Bangladesh; 4 Department of International Health, Institute of Tropical Medicine (NEKKEN) and the Global Center of Excellence programme, Nagasaki University, Japan; 5 Department of Molecular Microbiology and Immunology, John Hopkins Bloomberg School of Public Health, Baltimore, MD 21205, USA

## Abstract

**Background:**

Malaria is a major public health burden in Southeastern Bangladesh, particularly in the Chittagong Hill Tracts region. Malaria is endemic in 13 districts of Bangladesh and the highest prevalence occurs in Khagrachari (15.47%).

**Methods:**

A risk map was developed and geographic risk factors identified using a Bayesian approach. The Bayesian geostatistical model was developed from previously identified individual and environmental covariates (p < 0.2; age, different forest types, elevation and economic status) for malaria prevalence using WinBUGS 1.4. Spatial correlation was estimated within a Bayesian framework based on a geostatistical model. The infection status (positives and negatives) was modeled using a Bernoulli distribution. Maps of the posterior distributions of predicted prevalence were developed in geographic information system (GIS).

**Results:**

Predicted high prevalence areas were located along the north-eastern areas, and central part of the study area. Low to moderate prevalence areas were predicted in the southwestern, southeastern and central regions. Individual age and nearness to fragmented forest were associated with malaria prevalence after adjusting the spatial auto-correlation.

**Conclusion:**

A Bayesian analytical approach using multiple enabling technologies (geographic information systems, global positioning systems, and remote sensing) provide a strategy to characterize spatial heterogeneity in malaria risk at a fine scale. Even in the most hyper endemic region of Bangladesh there is substantial spatial heterogeneity in risk. Areas that are predicted to be at high risk, based on the environment but that have not been reached by surveys are identified.

## Background

Malaria is estimated to be responsible for one million deaths globally and 500 million clinical episodes in each year [1]. It remains an important public health problem in Bangladesh where it is mostly seasonal with its major incidence during the rainy season. Recent population based surveys indicate that malaria is endemic in 13/64 administrative districts and the crude prevalence is 4.0%. Most infections are due to *P. falciparum *(90.2%), *P. vivax *and co-infection with these two species (5.3 and 4.5% respectively). Even within this region malaria predominantly occurs within the Chittagong hill tracts. The Chittagong hill tracts consist of three hill districts (Rangamati, Bandarban and Khagrachari). Average prevalence in these three districts is 11.7%.

**Figure 1:**
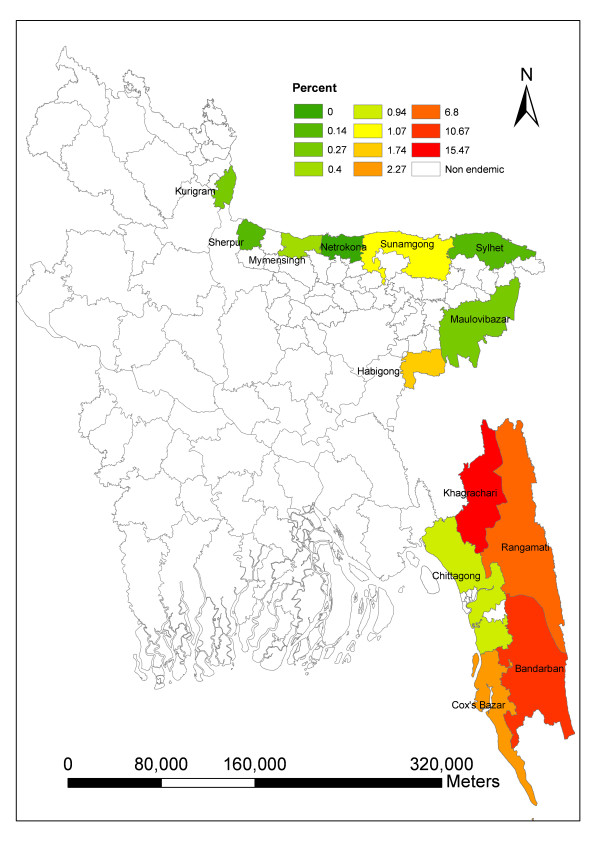
**Distribution of malaria prevalence in endemic areas of Bangladesh**.

Khagrachari has the highest prevalence in the endemic region (Fig. 1; 15.5%) [2] and *P. falciparum*, (14.8%) accounts for nearly all the infections -- *P. vivax *and mixed infections represent only 0.4% and 0.3% of infections, respectively. Thus, the current study focused on factors associated with risk of infection in this relatively homogeneous, high risk portion of the country. Khagrachhari covers 2699.6 sq km. The district consists of 8 upazilas (sub-districts) and the total population is 524,961 with the majority of the people identified as tribal. The district is mostly hilly and covered with forest [3]. The average annual temperature ranges between 13°C - 34.6°C and the annual rainfall 3031 mm.

In 2006, Bangladesh received $36.9 million USD from GFATM (Global Fund to Fight AIDS, Tuberculosis and Malaria) to control malaria in the 13 endemic districts. Large-scale initiatives were undertaken by BRAC in collaboration with the national ministry of health to implement a malaria control program. The programme integrated rapid diagnosis tests (RDTs), new drug regimens (Artemesinin-based combination therapy (ACT)), expanded distribution of LLIN (long lasting insecticide net), enhanced infection surveillance, vector surveillance and better documentation of activities.

Artemether-lumefantrine (Coartem) was adopted as a first-line treatment of *P. falciparum *malaria and a goal was to provide early diagnosis and prompt treatment to 80% of malaria patients. Other intervention objectives included effective malaria prevention for 80% of the population at risk and a strengthened epidemiological surveillance system. Surveys indicated that 40% of the households in high risk areas had nets, 10% of which were insecticide treated. Through this grant, it was expected that 80% of households (1.7 million) would be covered with LLINs. Nets currently present in households would be treated and re-treated twice a year with insecticide [4].

Malaria distribution maps are a strategy to target resource distribution and to focus the control program. In the absence of an active surveillance system, where surveillance is based on passive case detection, sample-based spatial prediction incorporating spatially varying covariates may be the best approach to target control initiatives [5,6]. With appropriate analyses the malaria risk map can extrapolate predicted risk from surveyed locations to unsampled locations.

These analyses typically incorporate spatially explicit data manipulation within a geographic information system (GIS), coupled with remote sensing (RS) to characterize environmental conditions and Bayesian spatial analysis to model the relationship between predictors and malaria risk [7]. Such model-based geostatistical approaches have recently been used to study the geographical distribution of tropical diseases both at larger or smaller scale including malaria [7]. These approaches were integrated to develop a malaria risk map for this highly endemic region of malaria. The goals were to identify a population level sampling frame, to identify individual and environmental correlates of risk and represent the spatial heterogeneity of risk so that intervention strategies could be evaluated and monitored.

## Methods

### Sample size selection, data collection and data preparation

Needed sample sizes were calculated using web-based software (C-Survey 2.0). Conservative estimates of malaria prevalence (2%), design effect (2), and precision (1.5% at 95% confidence interval) were used.

The study in Khagrachari was conducted in September, 2007. For Khagrachari, all mauzas (the lowest administrative unit of Bangladesh that has a polygon boundary) were listed and 30 mauzas were selected using a probability proportional to size (PPS) sampling procedure. The population figures from 2001 population census of Bangladesh were used for sampling [8] and a multi-stage cluster sampling technique was used. Twenty-five households were selected using systematic randomization from each mauza. The coordinates (longitude and latitude) of all selected households (n = 750) were recorded on-site using eTrex venture single handheld global positioning system (GPS) receivers. Simple random sampling was used to select one individual from each household. Economic status data was collected from the selected household when malaria prevalence survey was conducted. All age groups were eligible to participate and there were no sex discrimination.

Ethical approval was obtained from ICDDR,B ethical review committee. After obtaining written consent from the individual or their legal guardian, blood was collected from individual. Individuals were screened using rapid diagnostic test (RDT, FalciVax) to detect *Plasmodium falciparum *and *Plasmodium vivax*-specific antigens [9]. Standardization of this test was performed by Zephyr Biomedicals. Sensitivity and specificity of the RDT is reportedly more than 95% [9].

Environmental information was considered in this study [9]. Forest data was obtained from GeoNetwork world's forest data 2000 [10]. The forest cover resolution was 1 × 1 kilometer. A (ninety meter resolution) digital elevation model (DEM) from shuttle radar topographic mission [11] (SRTM) was used to obtain altitude data.

### Variable screening and selection

A malaria risk map of the study area was constructed in GIS via model based predictions. Bayesian geostatistical models were developed in WinBUGS 1.4 (Medical Research Council, Cambridge, UK and Imperial College London, UK). Covariates were selected using bivariate logistic regression derived from a larger socio-economic and demographic dataset collected during the national malaria baseline survey.

The Bayesian geostatistical model was developed from covariates that tended to be associated with malaria risk in the national survey (p < 0.2; economic status, age, forest types, and elevation). Household economic status was recorded as a qualitative variable i) all the year deficient ii) deficient sometimes iii) neither deficient nor surplus iv) surplus. Age ranged between 1 to 83 years (mean age = 28) for people in Khagrachari. Forest cover was related to the degree and pattern of clearing; closed forest, fragmented forest, or woodlot. Elevation (altitude of every household) data were considered for inclusion in the model. Bivariate logistic regression was performed on the data for Khagrachari using Stata version 10.1 (Stata Corporation, College Station, TX) and variables identified from the national survey with p > 0.2 in this region were excluded for further analysis. Other individual level and household variables such as bed net numbers and use, educational status, and knowledge of malaria transmission were initially considered for this analysis but were excluded later because they were not significantly associated with malaria infection in this study.

### Bayesian geostatistical prediction

Spatial autocorrelation was estimated within a Bayesian framework based on a geostatistical model. The individual infection status is considered a binary outcome variable *Y*_*i *_with *Y*_*i *_= 1 for infected individuals and 0 for non-infected individuals. The model assumed a conditional Bernoulli model for the binary outcome variable where the probability *p *of an individual *i *being infected, given the location *j *of the individual was:

where *Y*_*i*, *j *_is the infectious status of an individual in location *j*, *p*_*i*, *j *_is the probability of an individual being a case in location j, *α *is the intercept, *x*_*i*, *j *_is a matrix of covariates, *β *is a vector of coefficients and *u*_*i *_is a geostatistical random effect defined by an isotropic exponential spatial correlation function:

where *d*_*ab *_are the distances between pairs of points *a *and *b*, and *ϕ *is the rate of decline in the spatial correlation per unit distance. Non-informative priors were used for *α *(uniform prior with bounds -∞ and ∞) and the coefficients (normal prior with mean = 0 and precision = 1 × 10^-4^). The prior distribution of *ϕ *had a minimum of 1 and a maximum of 600 (phi ~ dunif(1, 600)). The precision of *u*_*i *_was given a non-informative gamma distribution (tau ~ dgamma(1,0.05)).

The prediction of the prevalence of infection was performed by *kriging *the geostatistical random effect and adding it to the sum of the products of the coefficients for the fixed effects and the values of the fixed effects at each prediction location. A burn-in of 5,000 iterations was used, followed by 14,000 iterations where values for the intercept, coefficients and predicted probability of infection at the prediction locations were stored. Diagnostic tests for convergence of the stored variables were undertaken, including visual examination of history and density plots; convergence was successfully achieved after 14,000 iterations. The outputs of Bayesian models including parameter estimates and spatial prediction are termed posterior distributions. These distributions fully represent uncertainties associated with estimated values. We summarized the posterior distributions in terms of the posterior mean and 95% Bayesian credible interval (CrI).

Maps of the posterior distributions of predicted prevalence were developed in a GIS (ArcView 9.2, ESRI, Redlands, CA). Samples of the posterior distributions of the coefficients from the model were used to produce prediction maps on a 0.05 × 0.05 decimal degree grid [7] covering the study area using the model estimates. Grid sizes were calculated according to computational limits. It was also considered to give a meaningful prediction density for the intervention. Surface interpolation was used in the GIS to produce the final map of the predicted prevalence.

Area under the curve (AUC) of the receiver operating characteristic was used to determine discriminatory performance of the model predictions relative to observed prevalence thresholds of 10% and 50%. An AUC value of 0.7 was used as an acceptable predictive performance [12].

For model validation the dataset of 750 locations was randomly partitioned into four groups. One group was sequentially omitted and used as the prediction file and the model was run for the remaining three groups.

## Results

**Figure 2:**
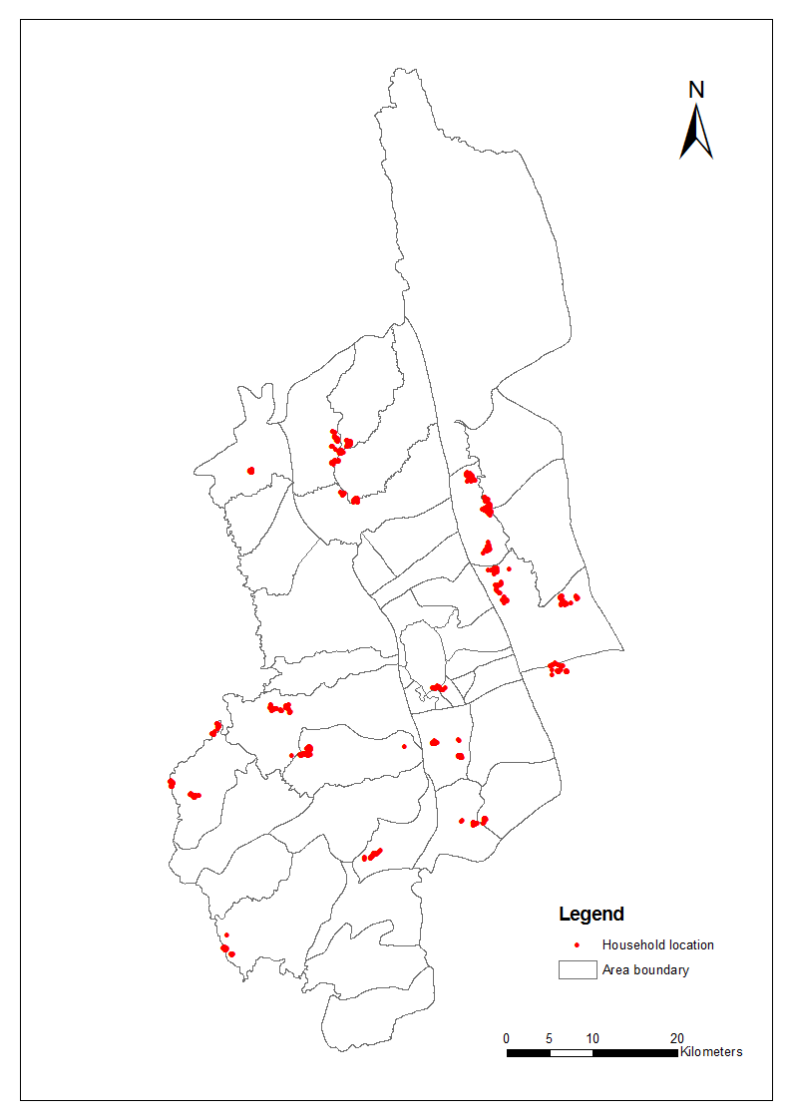
**Distribution of household locations in Khagrachari**.

The location of points where the current prevalence survey was conducted tended to be clustered and is a characteristic of the generally hilly conditions of the region where households have limited areas for placement (Fig. 2). Totally 750 individuals were screened from 750 individual houses. Crude prevalence rate was 15.47%. The prevalence rate was high among children but there was no discrimination in case of sex (Table 1). Highest prevalence occurred in households that reported neither deficient nor economic surplus but education, number of bed net did not prove significant risk factors (Table 1).

**Table 1:** Risk factors.

			Bivariate logistic regression		
**Variables**	**N (Population screened for RDT)**	**No. of malaria positives (%)**	**OR**	**95% CI**	**P-Value**

**Sex**					
Female	416	55 (16.47)	1		
Male	334	61 (14.66)	1.14	0.77 - 1.71	0.497
**Age**					
0-4	49	15 (30.61)	1		
5-14	154	48 (31.17)	1.03	0.51 - 2.06	0.942
15-49	436	46 (10.55)	0.27	0.14 - 0.53	0.001
≥ 50	111	7 (6.31)	0.15	0.06 - 0.41	0.001
**Education**					
No	347	58 (16.71)	1		
Yes	403	58 (14.39)	0.84	0.56 -- 1.24	0.381
**Economic status**					
All the year deficient	126	16 (12.70)	1		
Deficient sometimes	268	37 (13.81)	1.10	0.59 - 2.07	0.764
Neither deficient nor surplus	239	46 (19.25)	1.64	0.89 - 3.03	0.116
Surplus	117	17 (14.53)	1.17	0.56 - 2.44	0.677
**Number of bed net**					
≤ 2	87	14 (16.09)	1		
≥ 2	663	102 (15.38)	0.95	0.52 -- 1.74	0.864
**Forest**					
Woodlot	32	4 (12.50)	0.98	0.33 - 2.95	0.974
Fragmented forest	315	40 (12.70)	1		
Deep forest	403	72 (17.87)	1.5	0.98 - 2.27	0.059
**Altitude**					
1 - 44	199	25 (12.56)	1		
45 -- 54	193	31 (16.06)	1.33	0.75 - 2.35	0.323
55 -- 64	185	29 (15.68)	1.29	0.73 - 2.30	0.381
65+	173	31 (17.92)	1.52	0.86 - 2.69	0.151

Based on the model (Table 2), age of individuals and the extent of forest fragmentation were predictors of variation in infection likelihood after adjusting the spatial autocorrelation. Younger ages were at increased risk for infection and individuals living in fragmented forests were at significantly increased (82%) risk of infection compared with those living in unbroken forest. Improved economic status appeared to be associated with elevated risk though the effects were not statistically significant. Similarly, risk increased with elevation although in this region the effect was not significant. Phi (*φ*), the rate of spatial decay in autocorrelation was 399 (Table 1). After accounting for the effects of the covariates the radius of clusters was approximately 0.9 km (*φ *was measured in decimal degrees and 3/*φ *determined the cluster size; one decimal degree is approximately 120 km). The average AUC value of the four validation models was 0.79.

**Table 2:** Results of the Bayesian logistic regression model.

Variable	Posterior distribution
	
	OR (95% CI)
Age*	**0.95 (0.93,0.97)**

Economic status (live with deficiency)	1
Economic status (Deficient sometimes)	1.11 (0.50, 2.14)
Economic status (No deficient nor surplus)	1.70 (0.78, 3.23)
Economic status (Surplus)	1.37 (0.52, 2.90)
Forest type (deep forest)	1
Forest type (fragmented forest)	1.82 (1.02, 3.16)
Forest type (other woodland)	1.16 (0.20, 3.46)
Elevation*	1.17 (0.90, 1.51)
Intercept	0.31 (0.13, 0.63)

Rate of decay of spatial correlation^#^	399 (147.8, 587.2)
Variance of spatial random effect	0.62 (0.03,2.39)

*CI = Credible interval; SD standard deviation; Values for the fixed effects are odds ratios; note the odds ratios for age and elevation are on a common scale, where the variables were standardized to have a mean = 0 and standard deviation = 1. ^#^in decimal degrees.

**Figure 3:**
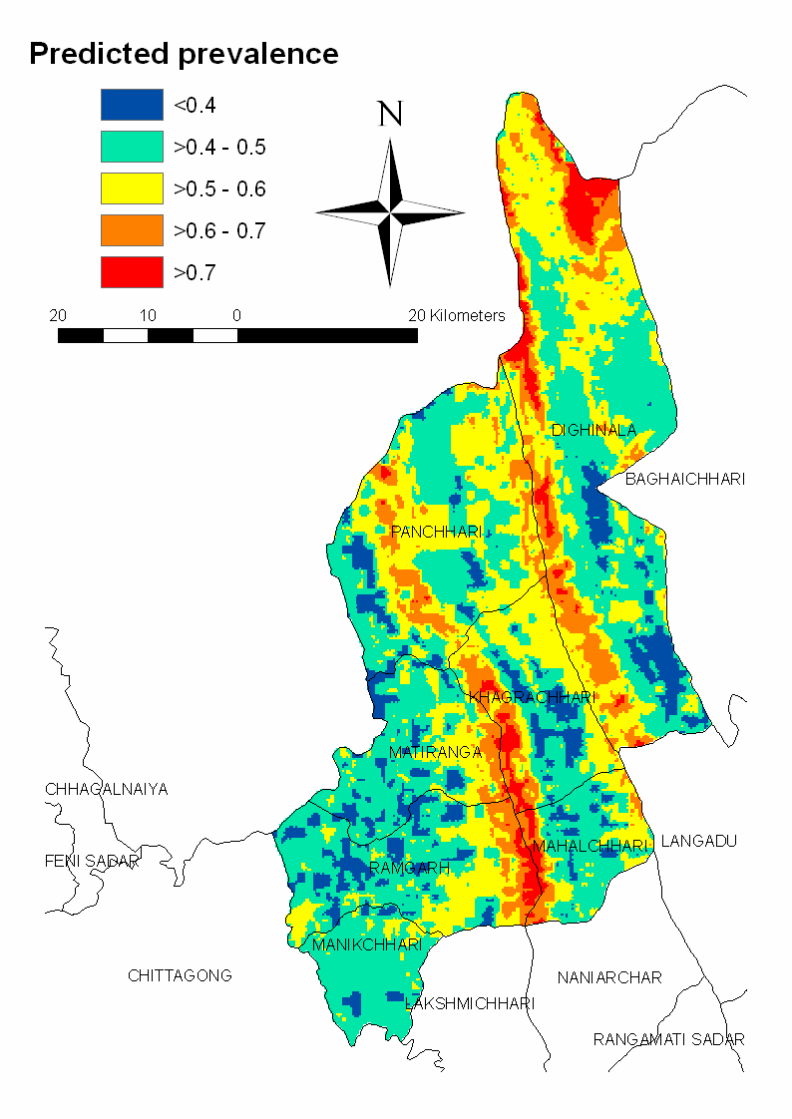
**Predicted malaria prevalence map in Khagrachari**.

**Figure 4:**
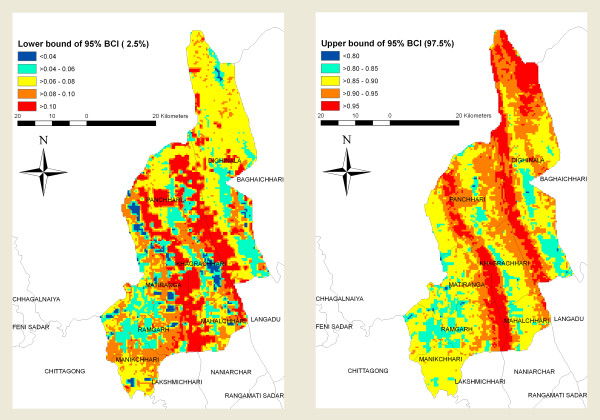
**The lower (left) and upper (right) percentiles of the posterior distribution for the predicted malaria prevalence**.

The predicted prevalence ranged from 31-84% (Figure 3). Site specific prevalence estimates, when mapped showed substantial geographic variability (Figure 3). Estimated rates were highest in the northeastern and central regions of the province and lowest in the southwestern and southeastern regions (Figure 3). The maps based on the boundaries of the 95%-CI of predicted malaria prevalence is presented in Figure 4.

## Discussion

The geostatistical model predicted that high prevalence areas were located along the north-eastern part and central area in Khagrachari along two river valleys. Except for Manikchari upazila, portions of every sub district were predicted to have large geographic extents of high risk. These regions were most widespread in Khagrachari and Dighinala upazilas.

The crude prevalence for the surveyed areas was 15.47%, which is consistent with the results of our previous study. However, this study indicates that the crude predicted prevalence across the region is 51%. This discrepancy could be due to the geographic coverage of previous surveys that had poor coverage in the highest risk areas (Figs. 2 and 4). Conversely, the precision of our predictions in areas where prevalence is above 50% are low, as indicated by the wide interval in the estimates (Fig. 3).

The different mean value indicate the transmission probability varied among different environmental conditions, as indicated by forest types, and age. Understanding the direct and indirect impact of these covariates is important. In the national malaria prevalence survey in Bangladesh prevalence was highest among children compared with older people [2] - a result confirmed in this study and similar to much of the published literature [13]. The lack of an effect associated with variation in economic status and altitude - variables that on a regional scale are significant was somewhat surprising. But, over this smaller, more homogeneous region where a high proportion of people live under poverty [14] and few people live in high altitude areas, the effects may not be detectable.

It was expected that areas of high risk would be widespread in Khagrachari as its selection was based on it being the highest endemic district in Bangladesh [2]. However, the analysis provides a more detailed, high resolution characterization for targeted implementation of control measures and programmatic evaluation. Targeting these hyper endemic areas at the sub-upazila level will become particularly important as Bangladesh scales up control operations.

Geostatistical tools of the spatial technology have helped revolutionize epidemiological research [15]. Maps provide an empirical basis to identify priority areas when implementing control and predicting the potential impact of control. At present there is a goal to distribute LLIN among 80% of households in endemic areas and retreatment for 40% of households' insecticide treated net (ITN) [4]. These analyses provide the background for a rational strategy to efficiently select those regions where resources are targeted so that the 80%/40% targets have the greatest impact on malaria infection.

## Conclusion

These findings represent an important strategy for targeting intervention and resources allocation. It can also be used as advocacy for directing funds to conduct more operational research in specific high risk areas. From a basic research perspective, identifying high risk malaria zones may generate new hypotheses regarding malaria transmission. Prediction of malaria risk with few covariates may compromise the detailed accuracy of the map. This is especially likely to occur when the geographic and sociological variability of the study area is small relative to the range of conditions in which the disease occurs.

## Competing interests

The authors declare that they have no competing interests.

## Authors' contributions

UH, RH, SMA and AI: designed and carried out the parasitological survey. UH conceived the study design and prepared the dataset for analysis. UH, RJSM, AC conducted geo-statistical analysis. UH wrote the manuscript. GG, RJSM, TY gave critical input and re-appraisal in the manuscript. All authors read and approved the final manuscript.
